# All-oral direct antiviral treatment for hepatitis C chronic infection in a real-life cohort: The role of cirrhosis and comorbidities in treatment response

**DOI:** 10.1371/journal.pone.0199941

**Published:** 2018-07-10

**Authors:** Noelle Miotto, Leandro Cesar Mendes, Leticia Pisoni Zanaga, Maria Silvia Kroll Lazarini, Eduardo Sellan Lopes Goncales, Marcelo Nardi Pedro, Fernando Lopes Goncales, Raquel Silveira Bello Stucchi, Aline Gonzalez Vigani

**Affiliations:** Internal Medicine Department, Infectious Diseases Division, Faculty of Medicine, State University of Campinas, Campinas, Brazil; Taipei Veterans General Hospital, TAIWAN

## Abstract

**Background:**

Hepatitis C virus (HCV) infection is the major cause of end-stage liver disease (LD) worldwide. The aim of this study was to assess sustained virological response (SVR) rates in a real-world cohort of patients with HCV infection treated with interferon-free direct antiviral agents (DAA).

**Patients and methods:**

All patients with genotypes 1, 2 or 3 HCV infection who started interferon-free treatment at a university hospital from December 2015 through July 2017 were included. The primary outcome was SVR at post-treatment week 12 by intention-to-treat (ITT) and modified ITT (mITT) analysis.

**Results:**

Five hundred twenty seven patients were enrolled, 51.6% with cirrhosis. Most patients received sofosbuvir + daclatasvir + ribavirin (60.7%) and sofosbuvir + simeprevir (25.6%). Overall SVR rates were 90.5% for ITT and 96% for mITT. SVR rates were higher in non-cirrhotic (94.2% in ITT and 96.8% in mITT) versus cirrhotic patients (87.1% in ITT and 95.2% in mITT). In ITT and mITT assessments, SVR rates were higher in patients with Child-Pugh A (n = 222, 88.7% and 95.7%, respectively) versus Child-Pugh B or C (n = 40, 80% and 90%, respectively); SVR rates were higher in patients with genotype 1 (n = 405, 92.1% and 98.2%), followed by genotype 2 (n = 13, 84.6% and 92.7%) and genotype 3 (n = 109, 84.4% and 88.4%). Lower comorbidity index (*p* = 0.0014) and absence of cirrhosis (*p* = 0.0071) were associated with SVR. Among cirrhotic patients, lower Model for End-Stage Liver Disease (*p* = 0.0258), higher albumin (*p* = 0.0015), and higher glomerular filtration rate (*p* = 0.0366) were related to SVR. Twenty-two cirrhotic patients (8%) had clinical liver decompensation during treatment. Complications of advanced LD were responsible for discontinuation of treatment and death in 12 and 7 patients, respectively.

**Conclusion:**

Treatment with all-oral DAA achieved high SVR rates, particularly in patients without cirrhosis and few comorbidities. Advanced LD is associated to poor outcome, such as treatment failure and death.

## Introduction

Hepatitis C virus (HCV) chronic infection affects 1.1% of the global population and is the leading cause of end-stage liver disease, hepatocellular carcinoma (HCC) and liver-related mortality in the Western world [1–3]. A sustained virologic response (SVR) after effective antiviral treatment is associated with decreased risk in liver disease progression and its complications, such as portal hypertension, hepatic decompensation, HCC, and liver transplantation [3–6]. Recently, treatment options for HCV infection and its efficacy have improved with the development of direct antiviral agents (DAA).

The polymerase inhibitor sofosbuvir (SOF), associated with the second-generation protease inhibitor (PI) simeprevir (SMV), or the NS5A inhibitor daclatasvir (DCV), with or without ribavarin (RBV), allowed interferon(IFN)-free effective regimens, with SVR rates above 90% in clinical trials [7–9]. However, those studies excluded or included few patients with advanced liver disease, so real-life studies comprising this population are needed. Furthermore, clinical trials also demonstrated variances in SVR rates between different genotypes, with lower SVR rates amongst genotype 3 cirrhotic patients [9–11]. Our study aimed to assess SVR rates and to identify underlying related factors in a large real-world cohort, including patients with advanced liver disease treated with IFN-free regimens.

## Materials and methods

### Patient enrolment

We included adult (> 18 years) patients with HCV chronic infection that started IFN-free DAA therapy at Clinic Hospital, State University of Campinas (UNICAMP), Brazil, from December 2015 through July 2017. HCV genotypes 1, 2, and 3 were included. Chronic HCV infection was defined as the presence of HCV antibody (Abott AxSYM Anti-HCV 3.0; Abbott Laboratories, Wiesbaden, Germany) and detectable serum HCV RNA (Cobas Ampli Prep Taq Man; Roche Diagnostics Systems Inc., Almere, The Netherlands). Treatment-naive patients and those who previously failed to PEG-IFN and RBV or to PEG-IFN and RBV plus first generation PI were included. We excluded patients with HIV infection, post-liver transplant, and those who previously received SOF, DCV or SMV.

### Stage of hepatic fibrosis evaluation

Stage of hepatic fibrosis was defined according to Metavir scoring system, transient hepatic elastography (Fibroscan^®,^ Echosense, Paris, France) or upon the combination of clinical and laboratorial parameters [12]. For analysis purposes, the diagnosis of none or minimal fibrosis was made upon histological examination (F0 or F1 stage) or liver stiffness (LS) under 7.1 kPa; portal fibrosis was defined as Metavir F2 or LS between 7.1 and 9.5 kPa: bridging fibrosis comprised histological stage F3 or LS between 9.5 and 12.5 kPa. The diagnosis of cirrhosis was made upon histological examination (F4 stage) or LS 12.5 kPa and / or the presence of esophageal varices, ascites, and splenomegaly [12–14].

### Treatment management and data collection

A questionnaire that included demographics, clinical characteristics and data about HCV infection was completed for each patient after medical appointment. The severity of medical conditions was estimated using Carlson’s comorbidity index (CCI) [15]. The estimation of glomerular filtration rate (eGFR) was performed using Modified Diet For Renal Disease [16]. Chronic kidney disease was classified according to the Kidney Disease Outcomes Quality Initiative criteria [17]. Clinical evaluation and laboratory tests were performed at baseline and every 4 weeks during treatment or more frequently, if needed. Serum biochemical and haematological analysis included haemoglobin (Hb), platelets, bilirubin, albumin, creatinine, aminotransferases, alaninotransferases, amylase, lipase, and prothrombin time. HCVRNA was performed at baseline, at treatment week 4, at the end of treatment (EOT) and post-treatment week 12 (PT12). Unquantifiable HCVRNA was defined as less than the lower limit of quantification. Among cirrhotic patients, Child-Pugh and Model for End-Stage Liver Disease (MELD) were calculated at baseline and at the EOT [18,19].

Safety was assessed by spontaneous adverse events (AE) reporting, by clinical evaluation and by laboratory data. Serious AE was defined as any AE that led to treatment discontinuation, decompensation of liver disease or grade 3 or 4 laboratory abnormalities. Mild anemia was defined as Hb 10.1–11.9 g/dL for women and Hb 10.1–12.9 g/dL for men; moderate and severe anemia was defined as Hb 8.6–10.0 g/dL and Hb ≤ 8.5 g/dL, respectively. Early therapy discontinuation was based on the decision of the physicians attending each patient. If treatment was interrupted by patients’ decision it was considered poor tolerability other than AE-related.

### Treatment dose and duration

Treatment was proposed to patients following standard practices and national guidelines at the outpatient clinic, without influence from the study team [20,21]. Genotype 1 patients with Child-Pugh B or C cirrhosis or prior non-responders to first generation PI-based treatment received SOF (400mg daily) plus DCV (60mg daily) with or without RBV for 24 weeks; the rest of genotype 1 patients received SOF plus DCV or SMV (150mg daily) with or without RBV for 12 weeks. Genotype 2 patients were treated with SOF plus RBV for 12 weeks. Genotype 3 patients received SOF plus DCV with or without RBV for 12 weeks. Ribavirin was adjusted by weight (1000mg/day for patients <75 kg and 1250mg/day for patients ≥ 75kg) and by glomerular filtration rate (eGFR). Changes in RBV dosages were documented, and DAA dosage did not change during treatment.

### Analysis population and endpoints

The treated population comprised all the patients that received at least 1 day of the purposed treatment. The primary endpoint was SVR, defined as unquantifiable HCVRNA at PT12. The primary analytic approach was an intention-to-treat (ITT) assessment. The secondary analytic approach was a modified intention-to-treat (mITT) assessment that excluded patients with missing virologic PT12 data due to loss to follow-up or death. Secondary endpoints comprised identification of factors associated with achievement of SVR and safety assessment.

Virologic failure was defined as absence of SVR due to no response (lack of achievement of unquantifiable HCVRNA during treatment), virologic breakthrough (quantifiable HCVRNA at EOT after an unquantifiable HCVRNA during treatment), or relapse (unquantifiable HCVRNA at EOT but quantifiable at PT12). In ITT assessment, non-virologic treatment failure included missing HCVRNA due to loss to follow-up or death on-or-after-treatment.

### Statistical analysis

We performed statistical analysis using Epi Info™, version 7.1.2.0 (Center for disease Control and Prevention, Atlanta, Georgia, USA) and GraphPad^®^(GraphPad Software, La Jolla, California, USA). Baseline continuous data were reported as median, and categorical values as frequencies and percentages. Univariate analyses were performed using 2- tailed Fisher’s, and analysis of variation or Mann-Whitney, as appropriate. A *p*<0.05 was considered statistically significant. Variables with *p*<0.2 were selected for a backward logistic regression model.

### Ethical considerations

Study design, protocols, patient enrolment, and data collection and storage were in accordance with ethical considerations supported by the Helsinki Declaration [22]. The study was reviewed and approved by the Ethics Committee for Research of the School of Medical Sciences, UNICAMP.

## Results

### Patients

**Fig 1:**
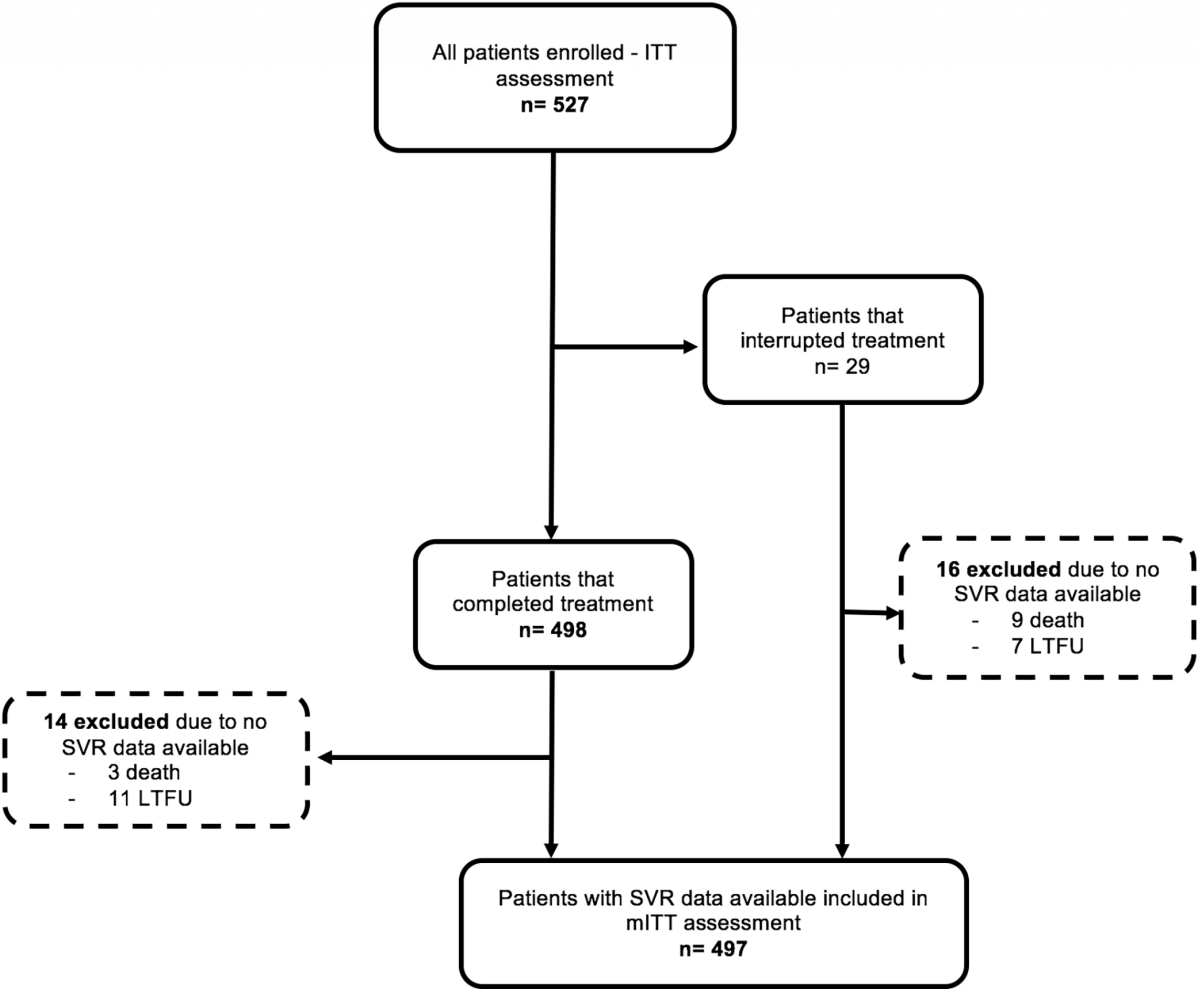
Derivation of the analysis population. ITT = intention-to-treat; LTFU = loss to follow-up; SVR = sustained virological response; mITT = modified intention-to-treat.

We included 527 patients treated with interferon-free DAA regimens, and 487 were included for mITT efficacy (Fig 1). Table 1 shows patients’ characteristics. Among all patients, median age was 56 years, most were male (59.8%), non-black (93.4%), and HCV-treatment-experienced (60.9%). Thirty-six patients (6.8%) had moderate chronic kidney impairment at baseline, and four patients were on haemodialysis. Cirrhosis was present in 51.6% of patients, most of them (81.6%) with compensated liver disease. Genotype 1 infection was the most prevalent (76.8%), followed by genotypes 3 (20.7%) and 2 (2.5%).

**Table 1:** Baseline characteristics of patients treated with all-oral direct antiviral agents, Campinas, Brazil (n = 526).

Parameter, *n* (%) unless otherwise indicated				All treated 527 (100%)	Genotype 1 405 (76.9%)		Genotype 3 109 (20.7)
**Demographics**							
	Age, year			56 (25–83)	55 (25–81)	63 (40–72)	57 (36–83)
	Male			315 (59.8)	252 (62.2)	6 (46.2)	57 (52.3)
	Race						
		Non-black		492 (93.4)	376 (92.8)	10 (77)	106 (97.2)
		Black		35 (6.6)	29 (7.2)	3 (23)	3 (2.8)
**Medical History**							
	Charlsson´s comorbidity index			5 (1–12)	5 (1–12)	4 (1–8)	5 (1–12)
	Stage of liver fibrosis^†^						
		None or minimal fibrosis		65 (12.3)	51 (12.6)	5 (38.5)	9 (8,.2)
		Portal fibrosis		108 (20.5)	86 (21.2)	2 (15.4)	20 (18.3)
		Bridging fibrosis		81 (15.4)	69 (17.0)	1 (7.6)	12 (11.1)
		Cirrhosis		272 (51.6)	199 (49.1)	5 (38.5)	68 (62.4)
		Child-Pugh A		222 (81.6)	159 (79.9)	5 (100)	58 (85.3)
		Child-Pugh B		37 (13.6)	31 (15.6)	-	6 (8.6)
		Child-Pugh C		13 (4.8)	9 (4.5)	-	4 (5.9)
		MELD		9 (6–22)	9 (6–22)	8 (7–10)	10 (6–18)
	HCV treatment-experienced			321 (60.9)	248 (61.2)	8 (61.5)	65 (59.6)
**Baseline laboratory values**							
	Albumin, g/dL			4.1 (2.1–5.1)	4.1 (2.1–5.1)	4.31(3.8–4.6)	4.1 (2.3–4.7)
	Bilirrubin, g/dL			0.81 (0.14–5.15)	0.82 (0.14–5.15)	0.62 (0.40–1.22)	0.85 (0.2–3.64)
	INR			1.11 (0.84–2.62)	1.10 (0.84–2.62)	1.11 (0.99–1.34)	1.14 (0.93–2.12)
	eGFR			90 (4–191)	91 (4–191)	83 (59–131)	90 (31–163)
	Hemoglobin, g/dL			14.6 (8.0–18.9)	14.7 (8.0–18.9)	14.0 (12.4–17.2)	14.4 (9.9–17.6)
	Platelets, 10^9^/L			156 (33–375)	156 (33–375)	211 (62–279)	155 (35–298)
	HCV viral load, log UI/mL			5.84 (2.97–7.31)	5.85 (2.97–6.44)	6.08 (4.18–6.90)	5.77 (3.07–7.31)
**Treatment regimens**							
	SOF + DCV + RBV		12 wk	202 (38.3)	113 (27.9)	89 (81.7)	-
			24 wk	118 (22.4)	118 (29.2)	-	-
	SOF + DCV		12 wk	35 (6.6)	15 (3.7)	20 (18.3)	-
			24 wk	13 (2.5)	13 (3.2)	-	-
	SOF + SMV + RBV		12 wk	11 (2.1)	11 (2.7)	-	-
	SOF + SMV		12 wk	135 (25.6)	135 (33.3)	-	-
	SOF + RBV		12 wk	13 (2.5)	-	-	13 (100)

Data presented as median and range, unless otherwise noted.

^†^One patient did not have evaluation of liver fibrosis and treatment was indicated because of extra hepatic manifestation.

MELD, Model for End-Stage Liver Disease; HCV, hepatitis C virus; INR, prothrombin international normalize ratio; eGFR, estimated glomerular renal function; SOF, sofosbuvir; DCV, daclatasvir; SMV, simeprevir; RBV, ribavirin; wk, weeks

Mean duration of treatment was 12 weeks (range 1–24). Table 1 illustrates treatment regimens and durations for each HCV genotype. Majority of patients received a combination of SOF + DCV + RBV (60.7%) followed by SOF + SMV (25.6%), and SOF + DCV (9.1%).

### Sustained virological response

SVR outcomes for ITT and mITT are shown in Table 2 for all patients, and broken down by genotype, cirrhotic status, and treatment regimens. Among all patients, SVR was 90.5% for ITT and 96% for mITT. SVR was higher in non cirrhotic patients (94.2% in ITT and 96.8% in mITT) compared to cirrhotic patients (87.1% in ITT and 95.2% in mITT). In both ITT and mITT assessments, SVR was higher in patients with cirrhosis Child-Pugh A (88.7% and 95.7%, respectively) than in patients with cirrhosis Child-Pugh Child B or C (80% and 90%, respectively).

**Table 2:** Sustained virologic response derived by genotype, cirrhosis status, HCV prior treatment, and treatment regimen, in intention-to-treat and modified intention-to-treat assessment (n = 527).

				Overall			Genotype 1				Genotype 2			Genotype 3			
				ITT (n = 527)	mITT (n = 497)		ITT (n = 405)	mITT (n = 381)	ITT (n = 13)		mITT (n = 12)	ITT (n = 109)		mITT (n = 104)
	SVR, n/N (%)			477/527 (90.5)	477/497 (96.0)	374/405 (92.1)	374/381 (98.2)	11/13 (84.6)	11/12 (92.7)	92/109) (84.4)	92/104 (88.4)
**Patients´ characteristics**											
	No cirrhosis			240/255 (94.2)	240/248 (96.8)	195/206 (94.6)	195/200 (97.5)	7/8 (87.5)	7/7 (100)	38/41 (92.1)	38/41 (92.1)
	Cirrhosis			237/272 (87.1)	237/249 (95.2)	179/199 (89.9)	179/181 (98.9)	4/5 (80.0)	4/5 (80.0)	54/68 (79.4)	54/63 (85.7)
		Child-Pugh A	197/222 (88.7)	197/206 (95.6)	145/159 (91.2)	145/147 (98.6)	4/5 (80.0)	4/5 (80.0)	48/58 (82.8)	48/54 (88.9)
		Child-Pugh B or C	40/50 (80.0)	40/43 (93.0)	34/40 (85.0)	34/34 (100)	-	-	6/10 (60.0)	6/9 (66.7)
	HCV—Treatment naive			182/206 (88.4)	182/191 (95.3)	143/157 (91.1)	143/146 (97.9)	4/5 (80.0)	4/4 (100)	35/44 (79.6)	35/41 (85.4)
	HCV—Treatment experienced			295/321 (91.9)	295/306 (96.4)	230/248 (92.7)	231/235 (98.3)	7/8 (87.5)	7/8 (87.5)	57/65 (87.7)	57/63 (90.5)
**Treatment regimen**											
	SOF + DCV + RBV		12 wk	176/202 (87.1)	176/185 (95.1)	101/113 (89.4)	101/101 (100)	NA		75/89 (84.3)	75/84 (89.3)
			24 wk	109/118 (92.4)	109/111 (98.2)	109/118 (92.4)	109/111 (98.2)	NA		NA	
	SOF + DCV		12 wk	32/35 (91.4)	32/35 (91.4)	15/15 (100)	15/15 (100)	NA		17/20 (85.0)	17/20 (85.0)
			24 wk	12/13 (92.3)	12/12 (100)	12/13 (92.3)	12/12 (100)	NA		NA	
	SOF + SMV + RBV		12 wk	10/11 (90.1)	10/10 (100)	10/11 (90.1)	10/10 (100)	NA		NA	
	SOF + SMV		12 wk	129/137 (94.2)	129/134 (96.3)	129/137 (94.2)	129/134 (96.3)	NA		NA	
	SOF + RBV		12 wk	11/13 (84.6)	11/12 (92.7)	NA		11/13 (84.6)	11/12 (92.7)	NA	

Data presented as median and range, unless otherwise noted.

HCV, hepatitis C virus; SVR, sustained virological response; ITT, intention-to-treat; mITT, modified intention-to-treat; SOF, sofosbuvir; DCV, daclatasvir; RBV, ribavarin; wk, weeks; SMV; simeprevir; NA, not applicable

In both ITT and mITT assessments, SVR was higher in patients infected with genotype 1 (n = 405, 92.1% and 98.2%), followed by a smaller group of genotype 2 (n = 13, 84.6% and 92.7%) and slightly lower in genotype 3 (n = 109, 84.4% and 88.4%).

Concerning the assorted treatment regimens for genotype 1- infected patients, SVR rates in ITT assessment for those treated with SOF + DCV + RBV for 12 and 24 weeks, and with SOF + SMV were 87.1% (176/202), 92.4% (109/118), and 94.2% (129/137), respectively. For patients with genotype 3, SVR rates were 84.3% (75/89) for patients treated with SOF + DCV + RBV, and 85% (17/20) for those who received SOF + DCV.

Regarding baseline characteristics among all patients in ITT assessment, lower CCI (*p* = 0.0014) and absence of cirrhosis (*p* = 0.0071) were associated with achievement of SVR. A sub-analysis in cirrhotic patients demonstrated that lower MELD (*p* = 0.0258), higher albumin (*p* = 0.0015), and higher eGFR (*p* = 0.0366) were related with SVR (Table 3.) There was no particular variable associated with SVR among non-cirrhotic patients. Multivariate analysis did not demonstrate any variable independently associated with SVR.

**Table 3:** Baseline characteristics associated with sustained virological response.

**All patients**			
*n* (%)	SVR, 477 (90.5)	No SVR, 50 (9.5)	*p*- Value
Age, years^‡^	56 (25–81)	57.5 (30–83)	0.1200
Sex, male (vs female)	282 (59.1)	33 (66.0)	0.3674
Race, non- black (vs black)	446 (93.5)	46 (92.0)	0.7628
Charlson Comorbidity Index^‡^	5 (1–11)	5.5 (1–12)	**0.0014**
Prior HCV tx, yes (vs no)	295 (61.8)	26 (52.0)	0.2225
Cirrhosis, yes (vs no)	237 (49.7)	35 (70.0)	**0.0071**
Ribavirin use, yes (vs no)	305 (63.9)	38 (76.0)	0.1180
eGFR, mL/min/m3^‡^	91 (4–191)	85.5 (33–173)	0.1884
Haemoglobin, g/dL^‡^	14.6 (8.0–18.9)	14.0 (9.4–18.2)	0.5469
HCVRNA, log^‡^	5.84 (3.07–7.44)	5.88 (2.97–7.15)	0.6717
**Cirrhotic patients**			
*n* (%)	SVR, 237 (87.1)	No SVR, 35 (12.9)	*p*- Value
Age, years^‡^	57 (29–81)	60 (30–83)	0.1610
Sex, male (vs female)	147 (62.0)	23 (65.7)	0.7128
Race, non- black (vs black)	221 (93.3)	32 (91.4)	0.7203
Charlson Comorbidity Index^‡^	5 (2–11)	6 (4–12)	0.2191
Prior HCV tx, yes (vs no)	156 (65.8)	21 (60.0)	0.5695
Ribavirin use, yes (vs no)	207 (87.3)	31 (88.6)	1.000
MELD^‡^	9 (6–22)	10.5 (6–22)	**0.0258**
Child-Pugh A (vs B or C)	197 (83.1)	25 (71.4)	0.1044
Albumin, g/dL^‡^	3.9 (2.1–5.0)	3.6 (2.5–4.7)	**0.0015**
Billirubin, g/dL^‡^	0.98 (0.14–4.40)	1.17 (0.20–5.15)	0.1054
INR^‡^	1.17 (0.89–2.12)	1.20 (0.98–2.23)	0.1885
eGFR, mL/min/m3^‡^	93 (4–191)	81.5 (33–173)	**0.0366**
Haemoglobin, g/dL^‡^	14.4 (8.0–18.9)	13.7 (9.4–17.1)	0.3886
Platelets, 10^9^/L^‡^	111 (33–360)	122 (38–375)	0.4763
HCVRNA, log^‡^	5.80 (3.07–7.34)	5.78 (2.87–6.92)	0.7907
**Non-cirrhotic patients**			
*n* (%)	SVR, 240 (94.1)	No SVR, 15 (5.9)	*p*- Value
Age, years^‡^	54 (25–81)	55 (36–79)	0.9019
Sex, male (vs female)	135 (56.3)	10 (66.7)	0.5927
Race, non- black (vs black)	225 (93.8)	14 (93.3)	1.0000
Charlson Comorbidity Index^‡^	4 (1–9)	4 (1–8)	0.3447
Prior HCV tx, yes (vs no)	139 (57.9)	5 (33.3)	0.1044
Ribavirin use, yes (vs no)	98 (40.8)	7 (46.7)	0.7880
eGFR, mL/min/m3^‡^	88 (4–180)	98.5 (59–116)	0.5078
Haemoglobin, g/dL^‡^	14.8 (9.3–18.5)	15.0 (12.8–18.2)	0.2507
HCVRNA, log^‡^	5.88 (3.18–7.44)	5.99 (4.23–7.15)	0.6717

^‡^Data shown in median and range. Bold values means statistically significant (p<0.05). SVR, sustained virological response; vs, versus; HCV, hepatitis C virus; eGFR, estimated glomerular filtration rate; MELD, model for end stage liver disease; INR, prothrombin international normalize ratio; tx = treatment.

### Treatment failure

Fifty patients on ITT assessment did not achieve SVR due to virologic (n = 18) or non-virologic (n = 32) failure. Among virologic failures, there were 1 null-responder 2 breakthroughs, and 15 relapses. Among non-virological failures, there were 2 patients that interrupted treatment before achieving a non-quantifiable HCVRNA; 12 patients died (4 during treatment and 8 during follow-up period); and 18 patients lost follow-up (6 during treatment and 12 after the EOT). Individual characteristics of the 50 patients with treatment failure are shown in Table 4. Among virologic failures, most patients (61.1%) were infected with genotype 3, 55.5% were HCV-previously treated, and half (n = 9) had cirrhosis. Concerning non-virologic failures, most patients had genotype 1 infection (75%), half (n = 16) were HCV-treatment experienced, and most were cirrhotic (59.3%).

**Table 4:** Baseline characteristics of patients with treatment failure.

#	Age	Sex	GT	Treatment Regimen	Actual Duration of Tx (weeks)	Prior HCV Tx (Y/N)	Cirrhosis (Y/N)	Child-Pugh Class	MELD Score	End of Treatment	Type of Failure
**Virologic Failure**											
1	55	M	1a	SOF + DCV + RBV 24 wk	24	Y	Y	A	9	Complete	Relapse
2	69	M	1	SOF + DCV + RBV 24 wk	24	Y	N	-	-	Complete	Breakthrough
3	56	F	1b	SOF + SMV 12 wk	12	Y	N	-	-	Complete	Null-responder
4	41	M	1b	SOF + SMV 12 wk	12	N	N	-	-	Complete	Relapse
5	79	F	1a	SOF + SMV 12 wk	12	N	N	-	-	Complete	Relapse
6	55	M	1b	SOF + SMV 12 wk	4	Y	N	-	-	Inter. AE	Relapse
7	63	M	2	SOF + RBV 12 wk	12	Y	Y	A	8	Complete	Relapse
8	58	F	3	SOF + DCV + RBV 12 wk	12	N	Y	A	15	Complete	Relapse
9	61	F	3	SOF + DCV + RBV 12 wk	12	Y	Y	C	18	Complete	Relapse
10	46	M	3	SOF + DCV + RBV 12 wk	12	N	Y	B	15	Complete	Relapse
11	52	M	3	SOF + DCV + RBV 12 wk	12	Y	Y	A	12	Complete	Relapse
12	56	M	3	SOF + DCV + RBV 12 wk	12	Y	Y	A	NA	Complete	Relapse
13	57	M	3	SOF + DCV + RBV 12 wk	12	Y	Y	5	9	Complete	Relapse
14	61	F	3	SOF + DCV 12 wk	12	N	Y	A	9	Complete	Relapse
15	36	F	3	SOF + DCV + RBV 12 wk	12	N	N	-	-	Complete	Breakthrough
16	52	M	3	SOF + DCV + RBV 12 wk	12	Y	N	C	18	Complete	Relapse
17	52	M	3	SOF + DCV + RBV 12 wk	12	N	N	-	-	Complete	Relapse
18	61	M	3	SOF + DCV 12 wk	12	N	N	-	-	Complete	Relapse
**Non-Virologic Failure**											
19	54	M	1a	SOF + DCV + RBV 24 wk	10	Y	Y	C	19	Inter. AE	Death
20	76	F	1b	SOF + DCV + RBV 12 wk	8	Y	Y	A	9	Inter. Death	Death
21	44	M	1b	SOF + DCV + RBV 12 wk	12	N	Y	A	8	Complete	LTFU
22	76	M	1a	SOF + DCV + RBV 24 wk	Unknown	Y	Y	A	9	LTFU	LTFU
23	45	M	1a	SOF + DCV + RBV 12 wk	12	N	Y	A	10	Complete	LTFU
24	55	M	1a	SOF + DCV + RBV 24 wk	Unknown	N	Y	C	22	LTFU	LTFU
25	60	M	1b	SOF + DCV + RBV 12 wk	12	N	Y	A	8	Complete	LTFU
26	64	F	1	SOF + DCV + RBV 24 wk	18	Y	Y	C	20	Inter. EA	Death
27	76	F	1b	SOF + DCV + RBV 12 wk	4	Y	Y	A	14	Inter. EA	Death
28	55	M	1a	SOF + DCV + RBV 12 wk	12	Y	Y	A	8	Complete	LTFU
29	30	M	1a	SOF + DCV + RBV 24 wk	11	Y	Y	A	12	Inter. EA	Death
30	47	F	1b	SOF + DCV + RBV 24 wk	4	Y	Y	C	21	Inter. EA	Death
31	41	F	1b	SOF + DCV + RBV 12 wk	Unknown	Y	Y	A	8	LTFU	LTFU
32	67	M	1b	SOF + DCV + RBV 24 wk	24	Y	Y	C	13	Complete	Death
33	78	F	1b	SOF + DCV + RBV 12 wk	12	N	Y	A	11	Complete	Death
34	65	M	1a	SOF + DCV + RBV 12 wk	8	Y	Y	5	8	Inter. AE	Death
35	65	F	1b	SOF + DCV + RBV 12 wk	12	N	Y	A	11	Complete	LTFU
36	45	M	1b	SOF + DCV + RBV 24 wk	24	N	Y	B	11	Complete	Death
37	76	F	1b	SOF + SMV 12 wk	4	N	Y	A	11	Inter. Intolerance	Non responder
38	55	M	1a	SOF + DCV + RBV 12 wk	Unknown	Y	N	–	-	LTFU	LTFU
39	45	M	1a	SOF + SMV + RBV 12 wk	12	N	N	-	-	Complete	LTFU
40	60	M	1b	SOF + DCV + RBV 12 wk	12	N	N	-	-	Complete	LTFU
41	66	F	1b	SOF + SMV 12 wk	12	Y	N	-	-	Complete	LTFU
42	40	M	1b	SOF + SMV 12 wk	Unknown	N	N	-	-	LTFU	LTFU
43	37	M	1	SOF + SMV 12 wk	Unknown	N	N	-	-	LTFU	LTFU
44	68	M	2	SOF + RBV 12 wk	12	N	N	-	-	Complete	LTFU
45	73	F	3	SOF + DCV + RBV 12 wk	4	Y	Y	A	6	Inter. Other	Non responder
46	57	M	3	SOF + DCV 12 wk	2	Y	Y	A	13	Inter. AE	Death
47	75	F	3	SOF + DCV + RBV 12 wk	8	N	Y	B	10	Inter. Other	Death
48	65	F	3	SOF + DCV + RBV 12 wk	1	N	Y	A	6	Inter. Other	LTFU
49	61	M	3	SOF + DCV + RBV 12 wk	12	N	Y	A	7	Complete	LTFU
50	83	F	3	SOF + DCV + RBV 12 wk	12	Y	Y	A	8	Complete	LTFU

GT, genotype; tx, treatment; HCV, hepatitis C virus; Y, yes; N, no; MELD, model for end stage liver disease; SOF, sofosbuvir; DCV, daclatasvir; RBV, ribavirin; wk, weeks; SMV, simeprevir; NA, not available; Inter, interrupted; AE, adverse events; LTFU, loss to follow-up.

### Safety

Forty-five (8.5%) patients experienced 1 or more serious AE. Mild anemia was seen in 33.9% (n = 179), moderate anemia in 5.5% (n = 29), and severe anemia in 1.7% (n = 9) of patients. Twenty-two cirrhotic patients (8%) had clinical liver decompensation during treatment.

Fifteen (2.8%) patients interrupted treatment due to AE: 12 due to liver decompensation, 2 due to sepsis and 1 due to severe anemia. Seven patients interrupted treatment because of non-AE causes: 4 because of intolerance; 1 due to dysphagia caused by ischemic stroke; 1 due to hepatocellular carcinoma -related liver transplant, and 1 for misunderstanding of correct medication dosage. There were 12 on-and-off-treatment deaths: 2 due to ischemic stroke (considered possibly related to treatment), 3 of sepsis, and 7 caused by complication of advanced liver disease (all with decompensated cirrhosis and 1 also with variceal bleeding). All death were classified as non-virologic treatment failure.

## Discussion

Our cohort comprised patients infected with diverse HCV genotypes and a high proportion of cirrhotic patients, including decompensated cirrhosis. We demonstrated high SVR rates in ITT assessment (90.5%), and even better in mITT (96%). SVR rates were higher among patients infected with genotype 1 and without cirrhosis. Among virologic failures, most patients had genotype 3 HCV-infection (63.6%) and half of them were cirrhotic.

Considering genotype 1-infected patients, SVR rates in our study (92.1% in ITT and 98.2% in mITT) were high and similar to those found in phase II Cosmos (92%), phase III OPTMIST-1 (97%), and phase III AI44040 (98%) clinical trials, even considering that those studies did not include or had few cirrhotic patients [7–9]. Our study had superior efficacy endpoint among patients that received SMV-based treatments (94.2% without RBV and 90.1% with RBV) compared to the TARGET cohort (84.2%) [23]. Moreover, we found that cirrhotic genotype 1 patients had lower SVR rate (89.9%) compared to non-cirrhotic patients (94.6%), which was also demonstrated by the HEPATHER study (87% and 98%, respectively) [24]. Our study included few patients with genotype 2 infection, consequently, we were not able to perform particular sub-analysis in this population. However, SVR rates (84.6% in ITT and 92.7% in mITT) among those patients were similar to another Brazilian cohort (88%) [25].

In our study, patients infected with genotype 3 had lower SVR rate compared to patients with genotypes 1 and 2. Efficacy outcome by ITT assessment for genotype 3 (84.4%) was slightly lower compared to the phase III studies ALLY-3 (89%) and ALLY-3+ (90%) [10,11]. This could be explained due to the low proportion of cirrhotic patients in the ALLY-3 (19.8%) compared to our study (62.4%). ALLY-3+ did not include decompensated cirrhosis and half of the patients received treatment for 16 weeks; while 15% of our cirrhotic patients had decompensated liver disease, and due to national guidelines, treatment duration was restricted to 12 weeks [10,11]. Among our findings, SVR in patients with genotype 3 and cirrhosis (79.4% in ITT and 85.7% in mITT) was somewhat lower than found among patients treated with SOF + DCV ± RBV the cirrhotic Spanish cohort (90.6 to100%), but comparable to the European compassionate study with 24 weeks duration treatment (88%), and to a Brazilian cohort (85%) [25–27]. We believe that genotype 3-infected patients, specially those with cirrhosis, are a difficult-to-treat populations that could benefit from treatment enlargement, as demonstrated in previous studies [26–27].

Prior studies revealed that HCV-treatment experienced patients achieved lower SVR rates [28,29]. Strikingly, in our study patients with prior HCV treatment had greater SVR rates (91.9% in ITT and 96.4% in mITT) compared to HCV-treatment naïve patients (88.4 in ITT and 95.3% in mITT). This was also demonstrated by the HEPATHER cohort, even for separate analysis between patients prior-null responders from prior relapsers and virologic breakthroughs. These results could be justified by different history of care and selection profiles, or even by compliance between treatment-experienced and treatment-naïve patients [24].

Besides cirrhosis status, we found that lower CCI index was associated with SVR (*p* = 0.0014). An Egyptian cohort showed that comorbidities were more frequent in patients with treatment failure (74.6%, p = 0.18), although CCI index was not performed [29]. Indeed, CCI index may be an important approach for individual patients before treatment. Higher CCI index is suitable with patients that need more attention while on-and- after treatment, due to the risk of drug-interactions and also treatment interruption [30–31].

Among cirrhotic patients, we demonstrated that higher albumin, lower MELD score and higher eGFR at baseline were associated with SVR achievement. Marcelin et al also showed that lower albumin was associated with treatment failure among patients with advanced fibrosis, and the TARGET cohort revealed that higher baseline albumin level was associated with SVR [23–28]. Although Child-Pugh A patients had superior SVR rate (88.7%) compared to Child-Pugh B and C (80%), Child-Pugh score was not an individual predictor of SVR achievement. Other previous studies also demonstrated that compensated cirrhotic patients had higher SVR rates compared to patients with decompensated liver disease, yet it was not statistically significant, except for one cohort that evaluated SVR among elderly patients [24,26,32]. Nevertheless, in our study lower MELD was independently associated with treatment response. Lastly, we found that higher eGFR was associated with SVR, which was not demonstrated by previous real-life studies[23,24,32]. Indeed, eGFR might be a confounded variable since it is included in MELD score. Although, higher eGFR could be associated with patients with a better health-status, explaining its association with SVR achievement. In despite of that, all the 4 patients with end-stage kidney disease included in our study achieved SVR12.

Our results showed that a small proportion of cirrhotic patients (8%) developed liver decompensation while on treatment. A British cohort including a large number decompensated cirrhotic patients (n = 409) demonstrated that 23% of those had worsening in MELD scores of 2 points or more [33]. Maan et al followed 433 cirrhotic patients treated with DAA and revealed that 11.5% of those experienced clinical liver decompensation, compared to 8% of cirrhotic patients in our study [34]. Decompensation of acute-on-chronic liver disease was also the main cause of treatment interruption due to AE (80%, n/N = 12/15) and death on-and-after treatment (58.3%, n/N = 7/12) in our casuistry. These data brings the attention to liver decompensation during treatment as an important cause of poor outcome.

Due to the observational nature of our study, no conclusion regarding superiority of one treatment regimen over another could be made. Also, genotype 1-infected patients with decompensated cirrhosis and those who previously failed from first-generation protease inhibitor received 24 weeks of DCV based-treatment, so groups that received 12 or 24 weeks of SOF + DCV ± RBV were not comparable. That said, no assessment between treatment duration could be done. Another important limitation of our study is that we do not have virologic analysis of failures. As most virologic failures were relapses rather than virologic breakthroughs and null-responders, we expect that treatment failures would be predominantly associated with resistance-associated variants [35]. Additional limitations of our study is missing data regarding Child-Pugh and MELD scores at EOT and the potential of under reporting of AE. However, it is unlikely that serious AE, which are clinically most relevant, were missed.

In conclusion, SVR rates amongst genotype 1 patients were high and similar to clinical trails and real-life cohorts, while SVR rates among genotype 3 patients were lower than those studies. Lower CCI index and absence of cirrhosis were associated with SVR achievement. Among cirrhotic patients, higher albumin, lower MELD and higher eGFR were related to treatment response. Nevertheless a small proportion of patients had liver decompensation, it was associated with poor outcome such as treatment interruption and death.
